# Methodology and development of a machine learning probability calculator: Data heterogeneity limits ability to predict recurrence after arthroscopic Bankart repair

**DOI:** 10.1002/ksa.12443

**Published:** 2024-09-26

**Authors:** Sanne H. van Spanning, Lukas P. E. Verweij, Laurent A. M. Hendrickx, Laurens J. H. Allaart, George S. Athwal, Thibault Lafosse, Laurent Lafosse, Job N. Doornberg, Jacobien H. F. Oosterhoff, Michel P. J. van den Bekerom, Geert Alexander Buijze, the Machine Learning Consortium, T. Flinkillä, S. Nakagawa, M. Loppini, B. R. Waterman, B. Owens, M. Gotoh, H. Nakamura, L. A. Rossi, I. Pasqualini, M. Scheibel, M. Minkus, J. S. Shaha, M. A. Ruiz Ibán, A. Barrow, R. T. Li, A. Lin, Y. V. Kleinlugtenbelt, J. M. Woodmass, P. MacDonald, J. Phadnis, A. Stone, C. Hatrick, T. P. van Iersel

**Affiliations:** ^1^ Alps Surgery Institute, Hand, Upper Limb, Peripheral Nerve, Brachial Plexus and Microsurgery Unit, Clinique Générale Annecy France; ^2^ Amsterdam Shoulder and Elbow Centre of Expertise (ASECE) Amsterdam The Netherlands; ^3^ Department of Human Movement Sciences Faculty of Behavioural and Movement Sciences, Vrije Universiteit Amsterdam, Amsterdam Movement Sciences Amsterdam The Netherlands; ^4^ Department of Orthopedic Surgery OLVG, Shoulder and Elbow Unit Amsterdam The Netherlands; ^5^ Amsterdam Movement Sciences, Musculoskeletal Health Program Amsterdam The Netherlands; ^6^ Department of Amsterdam UMC, Department of Orthopedic Surgery and Sports Medicine, Location AMC University of Amsterdam Amsterdam The Netherlands; ^7^ Department of Orthopaedic & Trauma Surgery Flinders Medical Centre, Flinders University Adelaide South Australia Australia; ^8^ Roth McFarlane Hand and Upper Limb Centre, Schulich School of Medicine and Dentistry Western University London Ontario Canada; ^9^ Department of Orthopaedic and Trauma Surgery, University Medical Center Groningen University of Groningen Groningen The Netherlands; ^10^ Department of Engineering Systems and Services Faculty Technology Policy and Management, Delft University of Technology Delft The Netherlands; ^11^ Department of Orthopedic Surgery, Montpellier University Medical Centre, Lapeyronie Hospital University of Montpellier Montpellier France; ^12^ Oulu University Hospital Oulu Finland; ^13^ Department of Orthopaedic Sports Medicine Yukioka Hospital Osaka Osaka Japan; ^14^ Humanitas Clinical and Research Center IRCCS, Rozzano Milan Italy; ^15^ Wake Forest University School of Medicine, Atrium Health Wake Forest Baptist Medical Center Winston‐Salem North Carolina USA; ^16^ Department of Orthopaedics, Warren Alpert Medical School Brown University Providence Rhode Island USA; ^17^ Department of Orthopedic Surgery Kurume University Medical Center Fukuoka Japan; ^18^ Institute of Orthopaedics “Carlos E. Ottolenghi” Hospital Italiano de Buenos Aires Buenos Aires Argentina; ^19^ Department of Orthopaedic Surgery Cleveland Clinic Foundation Cleveland Ohio USA; ^20^ Center for Musculoskeletal Surgery, Charité‐University Medicine Berlin Berlin Germany; ^21^ Department of Shoulder and Elbow Surgery Schulthess Clinic Zurich Switzerland; ^22^ Department of Orthopedic Surgery Mayo Clinic Phoenix Arizona USA; ^23^ Department of Orthopaedic Surgery and Traumatology Shoulder and Elbow Reconstructive Surgery Unit Madrid Spain; ^24^ UPMC Center for Sports Medicine Pittsburgh Pennsylvania USA; ^25^ Wake Orthopaedics, WakeMed Health and Hospitals Raleigh North Carolina USA; ^26^ Department of Orthopaedic Surgery Deventer Ziekenhuis Deventer the Netherlands; ^27^ Boston Shoulder Institute Boston Massachusetts USA; ^28^ Orthopedics, University of Manitoba Max Rady College of Medicine Winnipeg Manitoba Canada; ^29^ Brighton and Sussex Medical School, BSMS Teaching Building University of Sussex Brighton UK; ^30^ Department of Orthopaedic Surgery, OLVG Shoulder and Elbow Unit, Joint Research Amsterdam the Netherlands

**Keywords:** arthroscopic Bankart repair, artificial intelligence, dislocation, machine learning algorithm, recurrence, shoulder instability

## Abstract

**Purpose:**

The aim of this study was to develop and train a machine learning (ML) algorithm to create a clinical decision support tool (i.e., ML‐driven probability calculator) to be used in clinical practice to estimate recurrence rates following an arthroscopic Bankart repair (ABR).

**Methods:**

Data from 14 previously published studies were collected. Inclusion criteria were (1) patients treated with ABR without remplissage for traumatic anterior shoulder instability and (2) a minimum of 2 years follow‐up. Risk factors associated with recurrence were identified using bivariate logistic regression analysis. Subsequently, four ML algorithms were developed and internally validated. The predictive performance was assessed using discrimination, calibration and the Brier score.

**Results:**

In total, 5591 patients underwent ABR with a recurrence rate of 15.4% (*n* = 862). Age <35 years, participation in contact and collision sports, bony Bankart lesions and full‐thickness rotator cuff tears increased the risk of recurrence (all *p* < 0.05). A single shoulder dislocation (compared to multiple dislocations) lowered the risk of recurrence (*p* < 0.05). Due to the unavailability of certain variables in some patients, a portion of the patient data had to be excluded before pooling the data set to create the algorithm. A total of 797 patients were included providing information on risk factors associated with recurrence. The discrimination (area under the receiver operating curve) ranged between 0.54 and 0.57 for prediction of recurrence.

**Conclusion:**

ML was not able to predict the recurrence following ABR with the current available predictors. Despite a global coordinated effort, the heterogeneity of clinical data limited the predictive capabilities of the algorithm, emphasizing the need for standardized data collection methods in future studies.

**Level of Evidence:**

Level IV, retrospective cohort study.

**Abbreviations**
ABR: arthroscopic Bankart repair
ACL: anterior cruciate ligament
ALPSA: anterior labrum periosteal sleeve avulsion
AUC: area under the receiver operating curve
CI: confidence interval
GLAD: glenolabral articular disruption
GTF: greater tuberosity fracture
HAGL: humeral avulsion of the glenohumeral ligament
IGHL: inferior glenohumeral ligament lesion
ML: machine learning
OR: odds ratio
RCT: rotator cuff tear
ROC: receiver operator characteristic
SLAP: superior labrum from anterior to posterior
TRIPOD: transparent reporting of a multivariable prediction model for individual prognosis or diagnosis (TRIPOD)

## INTRODUCTION

Anterior shoulder dislocations are a common problem, with estimated rates in the United States of 0.24 per 1000 person‐years [30, 50]. Determining the most appropriate treatment (e.g., conservative treatment, labral repair such as arthroscopic Bankart repair [ABR] or a bone block procedure) is critical to maximize the chances of success [7, 8, 9, 48]. However, there is high heterogeneity and variation in evaluated risk factors between studies [42, 45]. Taking these factors into account during the decision‐making process therefore remains challenging. Several tools have been created to guide physicians in clinical decision‐making, such as the ISIS score, but these tools do not provide a patient‐specific probability [3]. Furthermore, the ISIS score prediction tool, which assigns points based on age with a cut‐off value of 20 years indicating a decreased risk of recurrent dislocation, is likely too simplistic. This is because the risk of recurrence significantly decreases beyond 20 years of age [11]. Recently, a prediction model was created to predict infection of tibial shaft fractures after intramedullary nailing based on patient‐specific factors using artificial intelligence [17]. This innovative tool enables physicians to enter risk factors into an online machine learning (ML) driven probability calculator and get a patient‐specific probability of infection. ML (i.e., artificial intelligence) has proven to be useful as clinical prediction models in several other orthopaedic studies [24, 27, 36]. As multiple events are needed per potential risk factor to acquire enough power for analysis, a large sample size is needed to build a patient‐specific algorithm to predict recurrent shoulder instability following a Bankart repair [2]. Therefore, an algorithm built on multicenter cohorts is necessary to maximize generalizability.

The aim of this study was to develop and train an ML algorithm to create a clinical decision support tool (i.e., ML‐driven probability calculator) to be used in clinical practice to estimate recurrence rates following an ABR.

## MATERIALS AND METHODS

### Guidelines

This study was performed according to the TRIPOD statement and the Guidelines for Developing and Reporting Machine Learning Predictive Models in Biomedical Research: A Multidisciplinary View [5, 16].

### Outcome measures and definitions

The primary outcome measure was recurrent shoulder instability following primary ABR without remplissage with a minimum follow‐up of 2 years. Shoulder instability was defined as either a complete dislocation or subluxation [1]. Remplissage was excluded to focus solely on evaluating primary ABR, as including remplissage would increase heterogeneity.

### Patient selection

Patients were selected from studies that evaluated risk factors for recurrence following ABR. Inclusion criteria were (1) patients treated with ABR without remplissage for traumatic anterior shoulder instability and (2) a minimum of 2 years follow‐up. Exclusion criteria include (1) patients who have undergone previous stabilization procedures and/or other surgical procedures than ABR to the ipsilateral shoulder and (2) patients with posterior, multidirectional or voluntary habitual instability. Relevant studies were identified up to September 2021 through a systematic approach searching the following databases using search terms described by Verweij et al.*:* PubMed, Embase/Ovid, Cochrane Database of Systematic Reviews/Wiley, Cochrane Central Register of Controlled Trials/Wiley, CINAHL/Ebsco, and Web of Science/Clarivate [45]. All authors of the relevant studies were contacted and asked to contribute by providing individual anonymized patient data. Ultimately, 14 out of 30 relevant studies provided their databases of patients treated between January 2000 and December 2020 (Table 1) [6, 14, 15, 19, 20, 31, 32, 33, 37, 38, 43, 47, 49].

**Table 1:** Characteristics of provided databases.

	Study	Author	Study design	Country	Samples collected between	Sample size (*n*)	Predictors	Min FU (mo)
1.	Long‐term results of arthroscopic Bankart repair: Minimum 10 years of follow‐up	Flinkkilä	Retrospective	Finland	2000–2005	186	Sex, age, number of anchors, SLAP, GLAD, RCT	120−192
2.	Risk Factors for the Postoperative Recurrence of Instability After Arthroscopic Bankart Repair in Athletes	Nakagawa	Retrospective	Japan	2010–2013	110	Sex, age, type of sport, Hill−Sachs	24
3.	Is the Instability Severity Index Score a Valid Tool for Predicting Failure After Primary Arthroscopic Stabilization for Anterior Glenohumeral Instability?	Loppini	Retrospective	Italy	2002–2009	670	Sex, age, type of sport, Hill−Sachs	62−157
4.	Outcomes After Bankart Repair in a Military Population: Predictors for Surgical Revision and Long‐Term Disability	Waterman	Retrospective	USA	2003–2010	3230	Sex, age, type of sport	24−84
5.	Risk factors for shoulder re‐dislocation after arthroscopic Bankart repair	Gotoh	Retrospective	Japan	2001–2011	102	Sex, age, type of sport, number of anchors, Hill−Sachs, HAGL, SLAP	25−120
6.	High Variability in Functional Outcomes and Recurrences Between Contact Sports After Arthroscopic Bankart Repair: A Comparative Study of 351 Patients With a Minimum 3‐Year Follow‐Up	Rossi	Retrospective	Argentina	2008–2016	304	Sex, age, type of sport, number of anchors, bony Bankart, Hill−Sachs, RCT	36−148
7.	Immobilization in External Rotation and Abduction Versus Arthroscopic Stabilization After First‐Time Anterior Shoulder Dislocation	Minkus	RCT	Germany	2011–2017	52	Sex, age, type of sport, bony Bankart, Hill−Sachs, RCT	24
8.	Redefining ‘Critical’ Bone Loss in Shoulder Instability	Shaha	Retrospective	USA	2009–2011	73	Sex, age, number of anchors	24−58
9.	Instability severity index score values below 7 do not predict recurrence after arthroscopic Bankart repair	Ruiz Ibán	Retrospective	Spain	2009–2012	113	Sex, age, type of sport, bony Bankart, ALSPA, SLAP, IGHL, GLAD, HAGL, Hill−Sachs, GTF, RCT	37−89
10.	On‐Track Lesions with a Small Distance to Dislocation Are Associated with Failure After Arthroscopic Anterior Shoulder Stabilization	Li	Retrospective	USA	2007–2015	193	Sex, age, Hill−Sachs, number of anchors	
11.	Original database (compiled from multiple articles)	Kleinlugtenbelt	Retrospective	The Netherlands	2005–2013	144	Sex, age, type of sport, Hill−Sachs, SLAP	24
12.	Postoperative Recovery Comparisons of Arthroscopic Bankart to Open Latarjet for the Treatment of Anterior Glenohumeral Instability	Woodmass	Retrospective	USA	2012–2017	50	Sex, age, type of sport, Hill−Sachs	24
13.	Utility of the Instability Severity Index Score in predicting failure after arthroscopic anterior stabilization of the shoulder	Phadnis	Retrospective	UK	2013–2020	84	Sex, age, type of sport, bony Bankart, ALSPA, SLAP, IGHL, GLAD, HAGL, Hill−Sachs, GTF, RCT, number of anchors	24−132
14.	Prognostic Factors associated with Failure to Return to Sport Following Primary Arthroscopic Bankart Repair: a Retrospective Multicenter Study	Iersel	Retrospective	The Netherlands	2014–2019	296	Sex, age, type of sport, bony Bankart, ALSPA, Hill−Sachs, GTF, RCT	29−72

Abbreviations: ALPSA, anterior labrum periosteal sleeve avulsion; FU, follow‐up; GLAD, glenolabral articular disruption; GTF, greater tuberosity fracture; HAGL, humeral avulsion of the glenohumeral ligament; IGHL, inferior glenohumeral ligament; mo, months; n, number; RCT, rotator cuff tears; SLAP, superior labrum from anterior to posterior.

### Data extraction

Variables that could be extracted from the fourteen provided databases included age, sex, type of sport, number of preoperative dislocations, anterior labrum periosteal sleeve avulsion (ALPSA), Bankart, glenolabral articular disruption (GLAD), Perthes, superior labrum from anterior to posterior (SLAP), greater tuberosity fracture, inferior glenohumeral ligament lesion (IGHL), humeral avulsion of the glenohumeral ligament (HAGL), bony Bankart and Hill−Sachs lesions, full and partial thickness rotator cuff tears (RCTs) and number of anchors. To create a high‐quality algorithm, data refinement was necessary to include as many patients as possible whilst including as many clinically relevant variables as possible, all of which had to be present in every patient included in the algorithm. This refinement was achieved through discussions among the authors. Four categories of sports were included: no sport/no contact, contact sport (defined as a sport where a person makes contact with people or objects constantly, with less force than collision sports including sports with high risk of hitting the ground or water with force, such as gymnastics and skiing), collision sport (defined as a sport where a person purposely hits or collides with other people or objects with great force) and sports with overhead throwing (defined as a sport where the person uses their upper arm and shoulder in an overhead movement to hit a ball toward the (opposing) team). Patients were classified in these different sports categories either by the authors providing the database or the first author (S.H.S.) of the current study. A bony Bankart was defined as a fracture of the glenoid involving the anterior labrum and glenoid rim [34]. A Hill−Sachs lesion was defined as an impression fracture of the posterolateral humeral head [34]. Subluxations were defined as the feeling of a dislocation that can be (spontaneously) reduced without the need for a radiographically confirmed dislocation [1].

### Identification of predictors using bivariate logistic regression analysis

To assess the association between predictors and recurrence, bivariate logistic regression analyses were performed. All variables provided by the authors with at least one event and non‐event were included (age, sex, type of sport, number of preoperative dislocations, ALPSA, Bankart, GLAD, Perthes, SLAP, HAGL, bony Bankart and Hill−Sachs lesions, full and partial thickness RCTs and number of anchors) to determine the association with recurrence. With ordinal variables, the highest value was used as reference in all the analyses. The bivariate logistic regression analyses were performed using Statistical Package for Social Sciences software (version 24, SPSS; IBM). A *p* value of <0.05 was considered significant.

### Development and internal validation of ML algorithms

The aim was to include at least 1000 patients in the algorithm, but this would lead to inclusion of only four variables with a total sample size of 1621 [44]. After careful consideration as described in ‘data extraction’, the variables included were sex, age at time of surgery, type of sport, bony Bankart, Hill−Sachs and number of pre‐operative dislocations and subluxations, leaving a total of 797 patients collected from four databases [31, 32, 33, 43]. The database was split into a train (80%, *n* = 638) and test data set (20%, *n* = 159) stratified on the outcome (recurrence) [4, 29]. To build the algorithm, first a random forest algorithm was used to assess the overall importance of the variables (identifying which variables contribute most to predicting the risk of recurrence). Then, four algorithms (support vector machine, neural network, Bayes point machine, and logistic regression) were developed and internally validated as prediction models using the six variables described above. These algorithms were chosen based on prior ML studies and their binary classification capabilities [46]. To train the algorithms in recognizing patterns related to recurrence following an ABR, 10‐fold cross‐validation was performed for each ML algorithm.

### Performance metrics

The predictive performance was assessed using the discrimination measured by the area under the receiver operator characteristic (ROC)‐curve, calibration (calibration slope, calibration intercept) and Brier score (overall model performance) [40]. The ROC curve plots the sensitivity (true positive rate) against 1 − specificity (false‐positive rate). The AUC ranges from 0.50 to 1.0 with 1.0 indicating the highest discriminating score and 0.50 indicating the lowest discriminating score. This differentiates between patients who had the outcome of interest (i.e., recurrence) from those who did not. Calibration was evaluated by analyzing the calibration slope and calibration intercept of a calibration curve. This assessment measures the association between the observed outcome and the predicted probability. A model with calibration intercepts of zero and calibration slopes of one is defined as a perfect model [39]. Overall performance—incorporating both discrimination and calibration—was assessed with the Brier score (zero is a perfect score and one is the lowest possible score) [40]. An extended description of the methods on the ML algorithms was previously published [44]. These statistical analyses were performed using R‐studio version 1.1.463 (R‐studio). The research fellow (L.H.) who performed these analyses was blinded to the origin of the data.

## RESULTS

### Patients

A total of 14 databases were provided and 5591 patients who received an ABR without remplissage were included. Databases originated from Europe, South and North America and Asia (Tables 1 and 2). Of the 5591 patients, 4797 were males (86%) and the mean age at surgery was 27 (ranged: 15–60, SD ± 7.8) years.

**Table 2:** Risk factors with corresponding number of databases, patient numbers and recurrence rates.

	Provided databases (*n*)	Patients (*n*)	Recurrence (%)
Age (y)	14	5591	15.4
Sex	14	5591	15.4
Female		794	13.7
Male		4797	15.6
Soft tissue lesions		5081	
ALSPA	4	471	14.7
Bankart	4	538	10.4
GLAD	3	381	20.7
IGHL	1	82	14.6
Perthes	3	470	14.7
SLAP	4	479	18.2
Bony lesions			
Bony Bankart	8	1776	15.1
GTF	4	540	10.4
HAGL	3	294	12.9
Hill‐Sachs	9	1952	15.9
Rotator cuff tear			
Full thickness	6	830	14.9
Partial thickness	5	725	14.6
Type of sport	12	5081	14.9
Contact		3752	16.0
Collision		338	15.4
Overhead throwing		204	7.4
No sport		787	11.0
Pre‐operative dislocations (*n*)	7	1054	13.2
0		156	12.2
1		286	8.0
2		135	16.3
3		78	20.5
4		122	14.8
≥5		277	14.8
Number of anchors (*n*)	4	435	18.2
1		1	0
2		19	21.1
3		217	20.3
4		171	15.8
≥5		27	14.8

Abbreviations: ALPSA, anterior labrum periosteal sleeve avulsion; GLAD, glenolabral articular disruption; GTF, greater tuberosity fracture; HAGL, humeral avulsion of the glenohumeral ligament; IGHL, inferior glenohumeral ligament; n, number; SLAP, superior labrum from anterior to posterior; y, years.

### Bivariate logistic regression analyses

According to the bivariate logistic regression analyses, age <35 years, participation in contact and collision sports, bony Bankart lesions and full‐thickness RCTs were associated with a higher risk of recurrence following an ABR (Table 3). One pre‐operative dislocation had a lower risk of recurrence. All other investigated variables did not demonstrate an association with recurrence following an ABR (Table 4). Three (HAGL lesion, greater tuberosity fracture and IGHL lesion) of the seventeen analyzed variables only had one event or nonevent and could therefore not be analyzed with bivariate logistic regression.

**Table 3:** Results of the bivariate logistic regression analyses for the different variables in relation to recurrence.

	Patients (*n*)	Recurrence (%)	*p* Value	OR	95% CI
Age (y)	5591				
15–19	691	20.1	<0.001	2.96	2.02−4.35
20–24	1804	18.3	<0.001	2.65	1.85−3.78
25–29	1274	16.0	<0.001	2.25	1.56−3.25
30–34	760	12.1	0.02	1.62	1.09−2.42
35–39	589	9.2	0.43	1.19	0.77−1.84
40–60	473	7.8	X	X	X
Sex	5591				
Female	794	13.7	0.18	1.16	0.93−1.44
Male	4797	15.6	X	X	X
Type of Sport	5081				
Contact	3752	16.0	<0.001	1.52	1.20−1.93
Collision	338	15.4	<0.05	1.46	1.01−2.11
Overhead throwing	204	7.4	0.12	0.64	0.36−1.12
No sport	787	11.0	X	X	X
Preoperative dislocations (*n*)	1054				
0	156	12.2	0.45	0.80	0.45−1.43
1	286	8.0	0.01	0.50	0.29−0.86
2	135	16.3	0.69	1.12	0.64−1.97
3	78	20.5	0.23	1.49	0.78−2.82
4	122	14.8	0.99	1.00	0.55−1.81
5	277	14.8	X	X	X
Soft tissue lesions					
ALSPA	469	14.7			
Present	63	6.4	0.05	0.36	0.13–1.02
Bankart	538	10.4			
Present	458	10.5	0.90	1.05	0.48–2.32
GLAD	381	20.7			
Present	10	10.0	0.41	0.42	0.05–3.34
Perthes	470	14.7			
Present	37	8.1	0.25	0.49	0.15–1.64
SLAP	479	18.2			
Present	79	17.7	0.91	0.97	0.51–1.81
Bony lesions					
Bony Bankart	1776	15.1			
Present	324	20.1	0.01	1.55	1.14−2.11
Hill‐Sachs	1952	15.9			
Present	1451	16.3	0.41	1.13	0.85–1.50
Rotator cuff tear					
Full thickness	830	14.9			
Present	25	40.0	<0.001	4.04	1.77–9.22
Partial thickness	725	14.6			
Present	16	18.8	0.64	1.36	0.38–4.85
Number of anchors (*n*)	435	18.2			
1	1	0.0	1.00	0.0	0
2	19	21.1	0.58	1.53	0.33–7.09
3	217	20.3	0.50	1.46	0.48–4.45
4	171	15.8	0.90	1.08	0.35–3.37
5	27	14.8	X	X	X

Abbreviations: ALPSA, anterior labrum periosteal sleeve avulsion; CI, confidence interval; GLAD, glenolabral articular disruption; n, number; OR, odds ratio; SLAP, superior labrum from anterior to posterior; X indicates reference value; y, years.

**Table 4:** Performance of machine learning algorithms in predicting recurrence (*n* = 797).

	Predictive performance			
	AUC	Calibration slope	Calibration intercept	Brier score
Trained algorithms				
Bayes point machine	0.57	0.49	−1.00	0.106
Logistic regression	0.54	0.46	0.46	0.106
Neural network	0.54	0.32	0.32	0.109
Support vector machine	0.54	0.36	0.36	0.106

Abbreviation: AUC, area under the receiver operative characteristics curve.

### ML algorithm

The algorithm was run with a total of 797 patients. The trained algorithms (Support Vector Machine, Neural Network, Bayes Point Machine and Logistic Regression) predicted recurrence following an ABR with an AUC ranging from 0.54 to 0.57. The predictive performance based on the AUC, calibration (calibration slope and calibration intercept) and Brier score is shown in Table 4.

## DISCUSSION

The most important finding of this study was that ML was not able to predict recurrence following ABR with the current available predictors. Despite a global coordinated effort, combining data sets was difficult due to the heterogeneity in definitions and used variables of the clinical data.

There are a few major advantages of ML algorithms above conventional scoring systems. First, these algorithms can consider a wide range of individual patient factors beyond simple demographic characteristics like age [21]. This allows for a more personalized assessment of recurrence risk, taking nuanced variations in patient characteristics, soft tissue and bony lesions, and other relevant variables into account. Second, unlike current scoring systems such as the ISIS score and the Nonoperative Instability Severity Index Score, ML algorithms are highly accurate in finding complex relationships and patterns within large data sets rather than relying on predefined rules or cutoffs [3, 10, 41]. They may therefore be more suitable in the analysis of risk factors potentially leading to more accurate predictions. Furthermore, ML does not use human‐selected statistical models. Rather, the algorithm is designed in a data‐driven manner, erasing human errors like picking the wrong statistical model [12]. Another major benefit of ML is the ability to learn from its own mistakes (incremental learning) during clinical use [25]. Data can be continuously added to improve its algorithm [25].

ML is becoming increasingly important in orthopaedics. Various models have been successfully designed, such as a model for identifying clinical features related to RCTs and the development of a predictive model for infection in tibial shaft fractures following intramedullary nailing [13, 17]. However, the effectiveness of ML algorithms may be limited compared to traditional logistic regression methods [26]. A study using data from the Norwegian Knee Ligament Registry (>60,000 patients) failed to develop a clinical calculator that is superior to previously created models with conventional statistics for the risk of revision surgery following anterior cruciate ligament (ACL) reconstruction [18]. One possible explanation suggested by the authors is the substantial amount of missing pre‐operative data. The current study also faced challenges due to poor data quality, including missing or incomplete information. Additionally, variability in the number of patients per variable (ranging from 82 to 5591) and the number of variables per patient (ranging from 3 to 30) required careful selection of databases for the development of the algorithm. Only four databases totalling 797 patients were included, falling short of the desired sample size for sufficient outcomes. Furthermore, important variables, such as the size of the bony Bankart and Hill−Sachs lesions and whether these lesions are on‐track or off‐track, could not be included despite their established association with an increased risk of recurrence [35].

Nevertheless, the previously mentioned advantages of ML algorithms above scoring systems in the treatment of shoulder instability highlight the need for further improvement of methodology for these studies. A practical guide, such as the one describe by Oeding et al. may guide physicians in the design and development of future ML models [22, 23]. However, to build large global data sets, it is essential to ensure that the data is suitable for pooling. To achieve this, a more universal agreement on what should be measured and how it should be measured has to be established. An important aspect in developing a robust algorithm is data labelling. This proved to be challenging in this study. As definitions differ extensively in current literature, it is plausible that there was a variation in definitions in the provided databases [1, 34]. This problem could be addressed by reaching a consensus on definition of a global Core Baseline Set. Within the present study, three variables were identified as predictors for recurrence following an ABR using a Random‐Forest algorithm. These predictors (number of pre‐operative dislocations, age at operation and Type of Sport) should therefore be integrated into a Core Baseline Set. In addition, a prior meta‐analysis identified bony lesions, ALPSA lesions and a surgical delay exceeding 6 months as additional risk factors that may be a valid addition to the Core Baseline Set [45]. Finally, what physicians consider to be important outcomes following treatment does not always correspond with what patients consider important [28]. Involving patients in defining what factors are important to investigate might improve the overall success rate of the treatment.

### Limitations

The current study must be interpreted within the context of several limitations. Firstly, the retrospective nature of data collection posed a significant challenge. As previously mentioned, the utilized databases exhibited variations in the investigated variables, and certain important variables, such as the size of glenoid bone loss and the size of Hill−Sachs lesions, could not be examined. Moreover, some variables were associated with only a single event, further complicating the analysis. To ensure the development of a reliable algorithm, it was imperative to include databases with identical variables. However, due to these discrepancies, only four databases encompassing a total of 797 patients could be incorporated into the algorithm, falling short of the intended number required for robust outcomes.

## CONCLUSION

Machine learning was not able to predict the recurrence following ABR repair with the current available predictors. Despite a global coordinated effort, the heterogeneity of clinical data limited the predictive capabilities of the algorithm emphasizing the need for standardized data collection methods in future studies.

## CONTRIBUTORS OF MACHINE LEARNING CONSORTIUM

T. Flinkillä (Oulu University Hospital, Oulu, Finland); S. Nakagawa (Department of Orthopaedic Sports Medicine, Yukioka Hospital, Osaka, Osaka, Japan); M. Loppini (Humanitas Clinical and Research Center, IRCCS, Rozzano, Milan, Italy); B. R. Waterman (Wake Forest University School of Medicine, Atrium Health Wake Forest Baptist Medical Center, Winston‐Salem, NC, USA); B. Owens (Department of Orthopaedics, Warren Alpert Medical School, Brown University, Providence, RI, USA); M. Gotoh (Department of Orthopedic Surgery, Kurume University Medical Center, Fukuoka, Japan); H. Nakamura (Department of Orthopedic Surgery, Kurume University Medical Center, Fukuoka, Japan); L. A. Rossi (Institute of Orthopaedics “Carlos E. Ottolenghi” Hospital Italiano de Buenos Aires, Buenos Aires, Argentina); I. Pasqualini (Department of Orthopaedic Surgery, Cleveland Clinic Foundation, Cleveland, OH, USA); M. Scheibel (Center for Musculoskeletal Surgery, Charité‐Universitaetsmedizin Berlin, Germany; Department of Shoulder and Elbow Surgery, Schulthess Clinic, Zurich, Switzerland); M. Minkus (Center for Musculoskeletal Surgery, Charité‐University Medicine Berlin, Berlin, Germany); J. S. Shaha (Department of Orthopedic Surgery, Mayo Clinic, Phoenix, AZ, USA); M. A. Ruiz Ibán (Shoulder and Elbow Reconstructive Surgery Unit, Department of Orthopaedic Surgery and Traumatology, Hospital Universitario Ramón y Cajal, Madrid, Spain); A. Barrow (UPMC Center for Sports Medicine, Pittsburgh, PA, USA); R. T. Li (Wake Orthopaedics, WakeMed Health and Hospitals, Raleigh, NC, USA); A. Lin (UPMC Center for Sports Medicine, Pittsburgh, PA, USA); Y. V. Kleinlugtenbelt (Department of Orthopaedic Surgery, Deventer Ziekenhuis, Deventer, the Netherlands); J. M. Woodmass (Boston Shoulder Institute, Boston, MA, USA); P. MacDonald (Orthopedics, University of Manitoba Max Rady College of Medicine, Winnipeg, MB, Canada); J. Phadnis (Brighton and Sussex Medical School, BSMS Teaching Building, University of Sussex, Brighton, UK); A. Stone (Brighton and Sussex Medical School, BSMS Teaching Building, University of Sussex, Brighton, UK); C. Hatrick (Brighton and Sussex Medical School, BSMS Teaching Building, University of Sussex, Brighton, UK); T. P. van Iersel (Shoulder and Elbow Unit, Joint Research, Department of Orthopaedic Surgery, OLVG, Amsterdam, the Netherlands).

## AUTHOR CONTRIBUTIONS

Sanne H. van Spanning: Initiation and protocol development, design, drafting or revising work, final approval, data acquisition and extraction, data analysis and interpretation. Lukas P. E. Verweij: Initiation and protocol development, design, drafting or revising work, final approval, data analysis and interpretation. Laurent A. M. Hendrickx: Drafting or revising work, final approval, data analysis and interpretation. Laurens J. H. Allaart: Drafting or revising work, final approval, design, data analysis and interpretation. George S. Athwal: Drafting or revising work, final approval, data analysis and interpretation. Thibault Lafosse: Drafting or revising work, final approval, data analysis and interpretation. Laurent Lafosse: Drafting or revising work, final approval, data analysis and interpretation. Job N. Doornberg: Drafting or revising work, final approval, data analysis and interpretation. Jacobien H. F. Oosterhoff: Drafting or revising work, final approval, data analysis and interpretation. Michel P. J. van den Bekerom: Initiation and protocol development, design, drafting or revising work, final approval, data analysis, interpretation. Geert Alexander Buijze: Initiation and protocol development, design, drafting or revising work, final approval, data analysis, interpretation and supervision. Sanne H. van Spanning, Lukas P. E. Verweij, Laurent A. M. Hendrickx, Laurens J. H. Allaart, George S. Athwal, Thibault Lafosse, Laurent Lafosse, Job N. Doornberg, Jacobien H. F. Oosterhoff, Michel P. J. van den Bekerom and Geert Alexander Buijze: Agreement to be accountable for all aspects of the work in ensuring that questions related to the accuracy or integrity of any part of the work are appropriately investigated and resolved.

## CONFLICT OF INTEREST STATEMENT

Dr. Laurens J. H. Allaart reports personal fees from Stryker, ConMed, Exatech, PercisionOS, Parvizi Surgical Innovation and Reach Orthopedics and has a patent pending to Exatech, ConMed and Stryker. Dr. Geert Alexander Buijze reports grants from SECEC, Vivalto Sante, personal fees from Depuy‐Synthes. Dr. Michel P. J. van den Bekerom reports grants for clinical and research fellowships supported by Smith and Nephew. Dr. Laurent Lafosse reports personal fees from Stryker, Smith and Nephew, Depuy. Dr. Thibault Lafosse reports personal fees from Stryker, Smith and Nephew, Depuy. The other authors have nothing to disclose. None of the fees above were related to the current study.

## Data Availability

The data that support the findings of this study are available on request from the corresponding author. The data are not publicly available due to restrictions from the original authors. Data can only be shared after obtaining agreement from the original authors who provided the data.

## References

[1] Alkaduhimi, H. , Connelly, J.W. , van Deurzen, D.F.P. , Eygendaal, D. & van den Bekerom, M.P.J. (2021) High variability of the definition of recurrent glenohumeral instability: an analysis of the current literature by a systematic review. Arthroscopy, Sports Medicine, and Rehabilitation, 3, e951–e966. Available from: 10.1016/j.asmr.2021.02.002 34195665 PMC8220632

[2] Austin, P.C. & Steyerberg, E.W. (2017) Events per variable (EPV) and the relative performance of different strategies for estimating the out‐of‐sample validity of logistic regression models. Statistical Methods in Medical Research, 26, 796–808. Available from: 10.1177/0962280214558972 25411322 PMC5394463

[3] Balg, F. & Boileau, P. (2007) The instability severity index score. A simple pre‐operative score to select patients for arthroscopic or open shoulder stabilisation. The Journal of Bone and Joint Surgery. British Volume, 89, 1470–1477. Available from: 10.1302/0301-620X.89B11.18962 17998184

[4] Breiman, L. (2001) Random forests. Machine Learning, 45, 5–32. Available from: 10.1023/A:1010933404324

[5] Collins, G.S. , Moons, K.G.M. , Dhiman, P. , Riley, R.D. , Beam, A.L. , Van Calster, B. , et al. (2024) TRIPOD+AI statement: updated guidance for reporting clinical prediction models that use regression or machine learning methods. BMJ, 385, e078378. Available from: 10.1136/bmj-2023-078378 38626948 PMC11019967

[6] Flinkkilä, T. , Knape, R. , Sirniö, K. , Ohtonen, P. & Leppilahti, J. (2018) Long‐term results of arthroscopic Bankart repair: minimum 10 years of follow‐up. Knee Surgery, Sports Traumatology, Arthroscopy, 26, 94–99. Available from: 10.1007/s00167-017-4504-z 28303281

[7] Griesser, M.J. , Harris, J.D. , McCoy, B.W. , Hussain, W.M. , Jones, M.H. , Bishop, J.Y. et al. (2013) Complications and re‐operations after Bristow‐Latarjet shoulder stabilization: a systematic review. Journal of Shoulder and Elbow Surgery, 22, 286–292. Available from: 10.1016/j.jse.2012.09.009 23352473

[8] Hovelius, L. & Rahme, H. (2016) Primary anterior dislocation of the shoulder: long‐term prognosis at the age of 40 years or younger. Knee Surgery, Sports Traumatology, Arthroscopy, 24, 330–342. Available from: 10.1007/s00167-015-3980-2 26754859

[9] Hurley, E.T. , Lim Fat, D. , Farrington, S.K. & Mullett, H. (2019) Open versus arthroscopic latarjet procedure for anterior shoulder instability: a systematic review and meta‐analysis. The American Journal of Sports Medicine, 47, 1248–1253. Available from: 10.1177/0363546518759540 29558168

[10] LeCun, Y. , Bengio, Y. & Hinton, G. (2015) Deep learning. Nature, 521, 436–444. Available from: 10.1038/nature14539 26017442

[11] Leland, D.P. , Bernard, C.D. , Keyt, L.K. , Krych, A.J. , Dahm, D.L. , Sanchez‐Sotelo, J. et al. (2020) An age‐based approach to anterior shoulder instability in patients under 40 years old: analysis of a US population. The American Journal of Sports Medicine, 48, 56–62. Available from: 10.1177/0363546519886861 31756127

[12] Ley, C. , Martin, R.K. , Pareek, A. , Groll, A. , Seil, R. & Tischer, T. (2022) Machine learning and conventional statistics: making sense of the differences. Knee Surgery, Sports Traumatology, Arthroscopy, 30, 753–757. Available from: 10.1007/s00167-022-06896-6 35106604

[13] Li, C. , Alike, Y. , Hou, J. , Long, Y. , Zheng, Z. , Meng, K. et al. (2023) Machine learning model successfully identifies important clinical features for predicting outpatients with rotator cuff tears. Knee Surgery, Sports Traumatology, Arthroscopy, 31, 2615–2623. Available from: 10.1007/s00167-022-07298-4 36629889

[14] Li, R.T. , Kane, G. , Drummond, M. , Golan, E. , Wilson, K. , Lesniak, B.P. et al. (2021) On‐track lesions with a small distance to dislocation are associated with failure after arthroscopic anterior shoulder stabilization. Journal of Bone and Joint Surgery, 103, 961–967. Available from: 10.2106/JBJS.20.00917 33764924

[15] Loppini, M. , Delle Rose, G. , Borroni, M. , Morenghi, E. , Pitino, D. , Domínguez Zamora, C. et al. (2019) Is the instability severity index score a valid tool for predicting failure after primary arthroscopic stabilization for anterior glenohumeral instability? Arthroscopy: The Journal of Arthroscopic & Related Surgery, 35, 361–366. Available from: 10.1016/j.arthro.2018.09.027 30611589

[16] Luo, W. , Phung, D. , Tran, T. , Gupta, S. , Rana, S. , Karmakar, C. et al. (2016) Guidelines for developing and reporting machine learning predictive models in biomedical research: a multidisciplinary view. Journal of Medical Internet Research, 18, e323. Available from: 10.2196/jmir.5870 27986644 PMC5238707

[17] Machine Learning Consortium, on behalf of the SPRINT and FLOW Investigators (2021) A machine learning algorithm to identify patients with tibial shaft fractures at risk for infection after operative treatment. Journal of Bone and Joint Surgery, 103, 532–540. Available from: 10.2106/JBJS.20.00903 33394819

[18] Martin, R.K. , Wastvedt, S. , Pareek, A. , Persson, A. , Visnes, H. , Fenstad, A.M. et al. (2022) Machine learning algorithm to predict anterior cruciate ligament revision demonstrates external validity. Knee Surgery, Sports Traumatology, Arthroscopy, 30, 368–375. Available from: 10.1007/s00167-021-06828-w 34973096 PMC8866372

[19] Minkus, M. , Königshausen, M. , Pauly, S. , Maier, D. , Mauch, F. , Stein, T. et al. (2021) Immobilization in external rotation and abduction versus arthroscopic stabilization after first‐time anterior shoulder dislocation: a multicenter randomized controlled trial. The American Journal of Sports Medicine, 49, 857–865. Available from: 10.1177/0363546520987823 33596092 PMC7961655

[20] Nakagawa, S. , Mae, T. , Sato, S. , Okimura, S. & Kuroda, M. (2017) Risk factors for the postoperative recurrence of instability after arthroscopic Bankart repair in athletes. Orthopaedic Journal of Sports Medicine, 5, 232596711772649. Available from: 10.1177/2325967117726494 PMC5593221 28959698

[21] Ngiam, K.Y. & Khor, I.W. (2019) Big data and machine learning algorithms for health‐care delivery. The Lancet Oncology, 20, e262–e273. Available from: 10.1016/S1470-2045(19)30149-4 31044724

[22] Oeding, J.F. , Williams, R.J. , Camp, C.L. , Sanchez‐Sotelo, J. , Kelly, B.T. , Nawabi, D.H. et al. (2023) A practical guide to the development and deployment of deep learning models for the orthopedic surgeon: part II. Knee Surgery, Sports Traumatology, Arthroscopy, 31, 1635–1643. Available from: 10.1007/s00167-023-07338-7 36773057

[23] Oeding, J.F. , Williams, R.J. , Nwachukwu, B.U. , Martin, R.K. , Kelly, B.T. , Karlsson, J. et al. (2023) A practical guide to the development and deployment of deep learning models for the orthopedic surgeon: part I. Knee Surgery, Sports Traumatology, Arthroscopy, 31, 382–389. Available from: 10.1007/s00167-022-07239-1 36427077

[24] Ogink, P.T. , Groot, O.Q. , Karhade, A.V. , Bongers, M.E.R. , Oner, F.C. , Verlaan, J.J. et al. (2021) Wide range of applications for machine‐learning prediction models in orthopedic surgical outcome: a systematic review. Acta orthopaedica, 92, 526–531. Available from: 10.1080/17453674.2021.1932928 34109892 PMC8519550

[25] Oosterhoff, J.H.F. & Doornberg, J.N. (2020) Artificial intelligence in orthopaedics: false hope or not? A narrative review along the line of Gartner's hype cycle. EFORT Open Reviews, 5, 593–603. Available from: 10.1302/2058-5241.5.190092 33204501 PMC7608572

[26] Oosterhoff, J.H.F. , Gravesteijn, B.Y. , Karhade, A.V. , Jaarsma, R.L. , Kerkhoffs, G.M.M.J. , Ring, D. et al. (2021) Feasibility of machine learning and logistic regression algorithms to predict outcome in orthopaedic trauma surgery. Journal of Bone and Joint Surgery, 104, 544–551. Available from: 10.2106/JBJS.21.00341 34921550

[27] Oosterhoff, J.H.F. , Karhade, A.V. , Oberai, T. , Franco‐Garcia, E. , Doornberg, J.N. & Schwab, J.H. (2021) Prediction of postoperative delirium in geriatric hip fracture patients: a clinical prediction model using machine learning algorithms. Geriatric Orthopaedic Surgery & Rehabilitation, 12, 215145932110622. Available from: 10.1177/21514593211062277 PMC8671660 34925951

[28] Park, I. , Kang, J.S. , Jo, Y.G. & Shin, S.J. (2019) Factors related to patient dissatisfaction versus objective failure after arthroscopic shoulder stabilization for instability. Journal of Bone and Joint Surgery, 101, 1070–1076. Available from: 10.2106/JBJS.18.01243 31220023

[29] Pavlov, Y. (2000) Random forests. Berlin, Boston: De Gruyter. Available from: 10.1515/9783110941975

[30] Paxton, E.S. , Dodson, C.C. & Lazarus, M.D. (2014) Shoulder instability in older patients. Orthopedic Clinics of North America, 45, 377–385. Available from: 10.1016/j.ocl.2014.04.002 24975764

[31] Phadnis, J. , Arnold, C. , Elmorsy, A. & Flannery, M. (2015) Utility of the Instability severity index score in predicting failure after arthroscopic anterior stabilization of the shoulder. The American Journal of Sports Medicine, 43, 1983–1988. Available from: 10.1177/0363546515587083 26122385

[32] Rossi, L.A. , Tanoira, I. , Gorodischer, T. , Pasqualini, I. & Ranalletta, M. (2020) High variability in functional outcomes and recurrences between contact sports after arthroscopic Bankart repair: a comparative study of 351 patients with a minimum 3‐year follow‐up. Arthroscopy, Sports Medicine, and Rehabilitation, 2, e575–e581. Available from: 10.1016/j.asmr.2020.07.004 33134997 PMC7588624

[33] Ruiz Ibán, M.A. , Asenjo Gismero, C.V. , Moros Marco, S. , Ruiz Díaz, R. , del Olmo Hernández, T. , Del Monte Bello, G. et al. (2019) Instability severity index score values below 7 do not predict recurrence after arthroscopic Bankart repair. Knee Surgery, Sports Traumatology, Arthroscopy, 27, 3905–3911. Available from: 10.1007/s00167-019-05471-w 30955072

[34] Rutgers, C. , Verweij, L.P.E. , Priester‐Vink, S. , van Deurzen, D.F.P. , Maas, M. & van den Bekerom, M.P.J. (2022) Recurrence in traumatic anterior shoulder dislocations increases the prevalence of Hill‐Sachs and Bankart lesions: a systematic review and meta‐analysis. Knee Surgery, Sports Traumatology, Arthroscopy, 30, 2130–2140. Available from: 10.1007/s00167-021-06847-7 34988633 PMC9165262

[35] Schwihla, I. , Wieser, K. , Grubhofer, F. & Zimmermann, S.M. (2023) Long‐term recurrence rate in anterior shoulder instability after Bankart repair based on the on‐ and off‐track concept. Journal of Shoulder and Elbow Surgery, 32, 269–275. Available from: 10.1016/j.jse.2022.07.025 36113705

[36] Shah, A.A. , Karhade, A.V. , Bono, C.M. , Harris, M.B. , Nelson, S.B. & Schwab, J.H. (2019) Development of a machine learning algorithm for prediction of failure of nonoperative management in spinal epidural abscess. The Spine Journal, 19, 1657–1665. Available from: 10.1016/j.spinee.2019.04.022 31059819

[37] Shaha, J.S. , Cook, J.B. , Song, D.J. , Rowles, D.J. , Bottoni, C.R. , Shaha, S.H. et al. (2015) Redefining “critical” bone loss in shoulder instability: functional outcomes worsen with “subcritical” bone loss. The American Journal of Sports Medicine, 43, 1719–1725. Available from: 10.1177/0363546515578250 25883168

[38] Shibata, H. , Gotoh, M. , Mitsui, Y. , Kai, Y. , Nakamura, H. , Kanazawa, T. et al. (2014) Risk factors for shoulder re‐dislocation after arthroscopic Bankart repair. Journal of Orthopaedic Surgery and Research, 9, 53. Available from: 10.1186/s13018-014-0053-z 24993404 PMC4105857

[39] Stevens, R.J. & Poppe, K.K. (2020) Validation of clinical prediction models: what does the “calibration slope” really measure? Journal of Clinical Epidemiology, 118, 93–99. Available from: 10.1016/j.jclinepi.2019.09.016 31605731

[40] Steyerberg, E.W. & Vergouwe, Y. (2014) Towards better clinical prediction models: seven steps for development and an ABCD for validation. European Heart Journal, 35, 1925–1931. Available from: 10.1093/eurheartj/ehu207 24898551 PMC4155437

[41] Tokish, J.M. , Thigpen, C.A. , Kissenberth, M.J. , Tolan, S.J. , Lonergan, K.T. , Tokish, Jr., J.M. et al. (2020) The nonoperative instability severity index score (NISIS): a simple tool to guide operative versus nonoperative treatment of the unstable shoulder. Sports Health: A Multidisciplinary Approach, 12, 598–602. Available from: 10.1177/1941738120925738 PMC7785891 32609577

[42] Trasolini, N.A. , Dandu, N. , Azua, E.N. , Garrigues, G.E. , Verma, N.N. & Yanke, A.B. (2021) Inconsistencies in controlling for risk factors for recurrent shoulder instability after primary arthroscopic Bankart repair: a systematic review. American Journal of Sports Medicine, 50, 3705–3713. Available from: 10.1177/036354652110387123635465211038712 34591717

[43] van Iersel, T.P. , Verweij, L.P.E. , Hoorntje, A. , Van der Hoeven, H. , Van Noort, A. , Kleinlugtenbelt, Y.V. et al. (2023) Prognostic factors associated with failure to return to sport following primary arthroscopic Bankart repair: a retrospective multicenter study. Journal of Shoulder and Elbow Surgery, 32, 1452–1458. Available from: 10.1016/j.jse.2023.01.003 36736656

[44] van Spanning, S.H. , Verweij, L.P.E. , Allaart, L.J.H. , Hendrickx, L.A.M. , Doornberg, J.N. & Athwal, G.S. et al. (2022) Development and training of a machine learning algorithm to identify patients at risk for recurrence following an arthroscopic Bankart repair (CLEARER): protocol for a retrospective, multicentre, cohort study. BMJ Open, 12, e055346. Available from: 10.1136/bmjopen-2021-055346 PMC9462090 36508223

[45] Verweij, L.P.E. , van Spanning, S.H. , Grillo, A. , Kerkhoffs, G. , Priester‐Vink, S. , van Deurzen, D.F.P. et al. (2021) Age, participation in competitive sports, bony lesions, ALPSA lesions, >1 preoperative dislocations, surgical delay and ISIS score >3 are risk factors for recurrence following arthroscopic Bankart repair: a systematic review and meta‐analysis of 4584 shoulders. Knee Surgery, Sports Traumatology, Arthroscopy, 29, 4004–4014. Available from: 10.1007/s00167-021-06704-7 34420117 PMC8595227

[46] Wainer, J. (2016) Comparison of 14 different families of classification algorithms on 115 binary datasets. CoRR abs/1606.00930.

[47] Waterman, B.R. , Burns, T.C. , McCriskin, B. , Kilcoyne, K. , Cameron, K.L. & Owens, B.D. (2014) Outcomes after Bankart repair in a military population: predictors for surgical revision and long‐term disability. Arthroscopy: The Journal of Arthroscopic & Related Surgery, 30, 172–177. Available from: 10.1016/j.arthro.2013.11.004 24485110

[48] Williams, H.L.M. , Evans, J.P. , Furness, N.D. & Smith, C.D. (2019) It's not all about redislocation: a systematic review of complications after anterior shoulder stabilization surgery. The American Journal of Sports Medicine, 47, 3277–3283. Available from: 10.1177/0363546518810711 30525905

[49] Woodmass, J.M. , Wagner, E.R. , Smith, J. , Welp, K.M. , Chang, M.J. , Morissette, M.P. et al. (2023) Postoperative recovery comparisons of arthroscopic Bankart to open Latarjet for the treatment of anterior glenohumeral instability. European Journal of Orthopaedic Surgery & Traumatology, 33, 1357–1364. Available from: 10.1007/s00590-022-03265-4 35665856

[50] Zacchilli, M.A. & Owens, B.D. (2010) Epidemiology of shoulder dislocations presenting to emergency departments in the United States. The Journal of Bone and Joint Surgery‐American, 92, 542–549. Available from: 10.2106/JBJS.I.00450 20194311

